# Supplementary material for: Sorting at embryonic boundaries requires high heterotypic interfacial tension
Source: Nat Commun. 2017 Jul 31;8:157. doi: 10.1038/s41467-017-00146-x (PMC5537356; doi:10.1038/s41467-017-00146-x)
Supplement: Supplementary file 2 — Supplementary Software 1 [file 41467_2017_146_MOESM2_ESM.zip › PottsModel/SrcPottsModel/doc/model/class-use/Cell.html]

Uses of Class model.Cell


JavaScript is disabled on your browser.


Skip navigation links


- Overview
- Package
- Class
- Use
- Tree
- Deprecated
- Index
- Help

- Prev
- Next

- Frames
- No Frames

- All Classes

## Uses of Class model.Cell

- Packages that use Cell

  | Package | Description |
  | --- | --- |
  |  |  |
  | --- | --- |
  | engine |  |
  | gui |  |
  | model |  |
- - ### Uses of Cell in engine

    Methods in engine with parameters of type Cell

    | Modifier and Type | Method and Description |
    | --- | --- |
    |  |  |
    | --- | --- |
    | `abstract java.lang.String` | CSVStatistic.`getCSVRow(PottsEngine paramPottsEngine, Cell paramCell)` |
    | `java.lang.String` | CellShapeCSVStatistic.`getCSVRow(PottsEngine pEngine, Cell pCell)` |
    | `java.lang.String` | CellCoordinatesCSVStatistic.`getCSVRow(PottsEngine pEngine, Cell pCell)` |
  - ### Uses of Cell in gui

    Constructors in gui with parameters of type Cell

    Constructor and Description

    `CellDisplay(Cell pCell)`
  - ### Uses of Cell in model

    Subclasses of Cell in model

    | Modifier and Type | Class and Description |
    | --- | --- |
    |  |  |
    | --- | --- |
    | `class` | `SquareCell` |

    Methods in model that return Cell

    | Modifier and Type | Method and Description |
    | --- | --- |
    |  |  |
    | --- | --- |
    | `Cell` | Pixel.`getCell()` |
    | `Cell` | CellSet.`getCell(int pCellID)` |

    Methods in model that return types with arguments of type Cell

    | Modifier and Type | Method and Description |
    | --- | --- |
    |  |  |
    | --- | --- |
    | `java.util.Iterator<Cell>` | Lattice.`getCellIterator()` |
    | `java.util.Iterator<Cell>` | CellSet.`iterator()` |

    Methods in model with parameters of type Cell

    | Modifier and Type | Method and Description |
    | --- | --- |
    |  |  |
    | --- | --- |
    | `void` | CellSet.`addCell(Cell pCell)` Adds a cell to the CellSet. |
    | `boolean` | CellSet.`contains(Cell pCell)` Function used when reading in the grid. |
    | `boolean` | Cell.`equals(Cell pOther)` |
    | `double` | Cell.`getDistanceToCell(Cell pCell)` |
    | `static double` | CellSet.`getLocalAreaEnergy(Cell pCell1, Cell pCell2)` Gets the area energy of the two cells passed as parameters. |
    | `static double` | Cell.`getLocalAreaEnergy(Cell pCell1, Cell pCell2)` The sum of the area-dependent energies. |
    | `static double` | CellSet.`getLocalPerimeterEnergy(Cell pCell1, Cell pCell2)` Gets the perimeter energy of the two cells passed as parameters. |
    | `static double` | Cell.`getLocalPerimeterEnergy(Cell pCell1, Cell pCell2)` The sum of the perimeter-dependent energies. |
    | `int` | Pixel.`getNumberOfNeighborsFromCell(Cell aCell)` |
    | `void` | CellSet.`removeCell(Cell pCell)` |

    Constructors in model with parameters of type Cell

    Constructor and Description

    `Cell(Cell pCell)`

    Copy constructor returning a copy of the passed BaseCell with identical
    member variables including a shallow copy of the pixelList.

    `Pixel(int pX,
    int pY,
    Cell pC,
    Lattice pL)`

    `Pixel(Pixel pPixel,
    Cell pC)`

Skip navigation links


- Overview
- Package
- Class
- Use
- Tree
- Deprecated
- Index
- Help

- Prev
- Next

- Frames
- No Frames

- All Classes
